# Soluble Vascular Cell Adhesion Molecule-1 (sVCAM-1) Is Elevated in Bronchoalveolar Lavage Fluid of Patients with Acute Respiratory Distress Syndrome

**DOI:** 10.1371/journal.pone.0149687

**Published:** 2016-02-26

**Authors:** Engi F. Attia, Sarah E. Jolley, Kristina Crothers, Lynn M. Schnapp, W. Conrad Liles

**Affiliations:** 1 Department of Medicine, Division of Pulmonary and Critical Care Medicine, University of Washington, Seattle, Washington, United States of America; 2 Department of Medicine, Section of Pulmonary/Critical Care and Allergy/Immunology, Louisiana State University Health Sciences Center, New Orleans, Louisiana, United States of America; 3 Department of Medicine, Division of Pulmonary, Critical Care, Allergy and Sleep Medicine, Medical University of South Carolina, Charleston, South Carolina, United States of America; 4 Departments of Medicine, Pathology, Pharmacology, and Global Health, Center for Lung Biology, University of Washington, Seattle, Washington, United States of America; French National Centre for Scientific Research, FRANCE

## Abstract

**Introduction:**

Pulmonary vascular endothelial activation has been implicated in acute respiratory distress syndrome (ARDS), yet little is known about the presence and role of endothelial activation markers in the alveolar space in ARDS. We hypothesized that endothelial activation biomarkers would be differentially expressed in bronchoalveolar lavage fluid from patients with ARDS compared with healthy volunteers, and that biomarker concentrations would be associated with ARDS severity.

**Methods:**

We performed a cross-sectional analysis of data from 26 intubated patients with ARDS undergoing evaluation for clinically suspected ventilator-associated pneumonia and five healthy volunteers. Patients underwent bronchoalveolar lavage a median of five days after intubation. Healthy volunteers also underwent bronchoalveolar lavage. Endothelial activation biomarkers (soluble vascular cell adhesion molecule-1 [sVCAM-1], soluble endothelial selectin [sESEL], angiopoietin-1 [Ang-1] and angiopoietin-2 [Ang-2]) were measured in bronchoalveolar lavage fluid. Clinically suspected ventilator-associated pneumonia was confirmed with microbiologic culture data.

**Results:**

Patients with ARDS had significantly higher median sVCAM-1 concentrations in the bronchoalveolar lavage fluid compared with healthy volunteers (985 vs 119 pg/mL, p = 0.03). Additionally, there was a trend toward greater bronchoalveolar lavage fluid sVCAM-1 concentrations among patients with moderate/severe compared to mild ARDS (1395 vs 209 pg/mL, p = 0.06). We did not detect significant differences in bronchoalveolar lavage fluid levels of sESEL, Ang-1 or Ang-2 between patients with ARDS and healthy volunteers. Median bronchoalveolar lavage fluid biomarker levels did not differ between patients with and without microbiologically-confirmed ventilator-associated pneumonia.

**Conclusions:**

sVCAM-1 concentrations were significantly higher in the bronchoalveolar lavage fluid of patients with ARDS compared to healthy controls, and tended to be higher in moderate/severe ARDS compared to mild ARDS. Our findings add to the growing evidence supporting the concept that endothelial activation plays an important mechanistic role in the pathogenesis of ARDS. Further studies are necessary to characterize the role and/or clinical significance of sVCAM-1 and other endothelial activation markers present in the alveolar space in ARDS.

## Introduction

Acute respiratory distress syndrome (ARDS) is a common cause of severe hypoxemia associated with mortality approaching 40% in U.S. hospitals [1]. ARDS is characterized by a robust inflammatory response encompassing both alveolar epithelial dysfunction and pulmonary vascular endothelial activation [2]. Pulmonary endothelial-epithelial barrier compromise contributes to increased vascular permeability and alveolar accumulation of inflammatory cells [3, 4]. Animal and *in vitro* models suggest that markers of endothelial activation may also accumulate in the alveolar space in ARDS [3, 5]. Whether a similar accumulation occurs in the lungs of mechanically ventilated patients with ARDS is unclear, and whether the presence of endothelial activation biomarkers in the alveolar space confers clinical significance is largely unknown.

Biomarkers of endothelial activation, such as endothelial selectin (ESEL) and vascular cell adhesion molecule-1 (VCAM-1) are expressed on the surfaces of activated human pulmonary microvascular endothelial cells [6], supporting that the cleaved, soluble forms of these molecules are relevant markers of endothelial activation in pulmonary pathology. Circulating serum/plasma soluble VCAM-1 (sVCAM-1) and angiopoietin-2 (Ang-2), another marker of endothelial activation, have been shown to be associated with ARDS [5, 7, 8]. However, studies measuring endothelial activation biomarkers in the bronchoalveolar lavage fluid (BALF) of patients with ARDS are limited and have yielded conflicting results. In one study, BALF soluble endothelial selectin (sESEL) was elevated among ARDS patients with chronic alcohol use [9]. Another study found higher Ang-2 and lower angiopoietin-1 (Ang-1) in the BALF of individuals with hyperoxia-induced acute lung injury [5]. A third demonstrated no difference in BALF soluble vascular cell adhesion molecule-1 (sVCAM-1) between patients with and without ARDS [8].

Additionally, there is a paucity of published data exploring the role of endothelial activation among patients with concomitant ARDS and ventilator-associated pneumonia (VAP). Up to 20% of patients receiving mechanical ventilation develop VAP, with greater frequency among patients with ARDS [10]. VAP is associated with greater organ dysfunction and higher mortality in severe ARDS [11, 12]. However, these studies primarily investigated epithelial cell activation, and limited data are available regarding the role of pulmonary endothelial activation on outcomes from ARDS and VAP.

Endothelial activation biomarkers in the alveolar space may be more specific for pulmonary, rather than systemic, inflammation and may provide insights into the pathogenesis of ARDS, particularly when associated with VAP. Therefore, in this study, we compared BALF concentrations of sVCAM-1, sESEL, Ang-1 and Ang-2 between patients with ARDS undergoing evaluation for VAP and healthy volunteers, and investigated whether biomarker concentrations were associated with ARDS severity. We hypothesized that these endothelial activation biomarkers would be differentially expressed in BALF from patients with ARDS compared to healthy volunteers, and that concentrations would differ by ARDS severity.

## Materials and Methods

### Cohort Study Participants

We performed a secondary analysis of data collected from adult intubated patients with ARDS undergoing bronchoscopy for suspected VAP. Bronchoscopy for diagnosis of clinically suspected VAP is standard practice at our institution, and these patients represented a convenience sample of ARDS patients cared for in the intensive care units of our institution in 2008 and 2009. ARDS was defined as: 1) PaO_2_/FiO_2_ <300 mmHg while receiving ≥5 cmH_2_O positive end-expiratory pressure, 2) diffuse parenchymal infiltrates, 3) pulmonary arterial wedge pressure <18 mmHg or lack of clinical evidence of congestive heart failure, and 4) no other obvious diagnosis explaining these findings. ARDS severity was based on the Berlin consensus definition as mild (200 mmHg < PaO_2_/FiO_2_ ≤ 300 mmHg) or moderate/severe (PaO_2_/FiO_2_ ≤200 mmHg) [13]. Clinical criteria for suspected VAP, based upon *American Thoracic Society/Infectious Disease Society of America* guidelines [14], included mechanical ventilation ≥48 hours, new or progressive pulmonary infiltrates on imaging, and ≥1 of the following: fever, leukocytosis or leukopenia, increased purulent endotracheal secretions, and no antibiotic changes for 72 hours. Control samples were obtained from healthy, non-smoking volunteers.

Written informed consent was obtained from participants or legal next of kin. Study procedures were approved by the Institutional Review Board at the University of Washington.

### BALF Collection

Participants with ARDS and healthy volunteers underwent protocolized bronchoscopy with BALF sample collection as previously described [15, 16]. Briefly, five separate 30 mL aliquots of 0.9% sterile saline were instilled in the most affected lobe radiographically, or the right middle lobe or left lingula, then aspirated and pooled. BALF was immediately centrifuged at 14,000 g for 20 minutes at 4°C and cell-free supernatant was aliquoted and stored at -80°C. Clinically suspected VAP was confirmed when bacterial cell culture grew ≥10^4^ colony forming units per milliliter (cfu/mL) or protected specimen brush culture grew ≥10^3^ cfu/mL [17].

### Biomarker Quantification

Concentrations of Ang-1, Ang-2, sESEL and sVCAM-1 were measured in aliquots of BALF supernatants using enzyme-linked sandwich immunoassay (ELISA), utilizing validated human-specific monoclonal antibody pairs (R&D Systems Duoset kits, Minneapolis, MN, USA) as previously described [18, 19]. Concentrations were extrapolated from simultaneously run standard curves.

### Clinical Data

Baseline clinical, physiologic and demographic data were obtained from the clinical electronic medical record for intubated patients with ARDS via standardized chart abstraction methods. Collected data included: duration of mechanical ventilation, PaO_2_:FiO_2_ ratios, microbiologic culture results, in-hospital mortality, age and gender.

### Statistical Analyses

Patient baseline characteristics were compared using Wilcoxon rank-sum tests for continuous and chi-square (χ^2^) or Fisher’s exact test for categorical variables. To account for non-parametric data, we used Wilcoxon rank-sum tests to compare median biomarker levels between patients with ARDS and healthy volunteers as well as by ARDS severity and microbiologically-confirmed VAP diagnosis. We repeated similar analyses restricted to patients without microbiologically-confirmed VAP as a sensitivity analysis to further evaluate the association of BALF endothelial activation biomarkers with ARDS independent of the effects of VAP. We also compared continuous values of BALF biomarkers with PiO2:FiO2 ratios, calculating the Pearson correlation coefficients (*r*). Finally, we compared biomarker levels with duration of mechanical ventilation and by in-hospital death, using Pearson correlation analyses and Wilcoxon rank-sum tests, respectively. A p-value of <0.05 was considered statistically significant. Analyses were performed in STATA version 13.1 (StataCorp LP, College Station, TX).

## Results

Baseline characteristics were compared between 26 patients with mild and moderate/severe ARDS (Table 1). Most patients were men (*n* = 16, 55%) with a median age of 65 years (*interquartile range [IQR*] 49–75). As expected, patients with mild ARDS had higher median PaO_2_:FiO_2_ ratios compared to patients with moderate/severe ARDS (220 mmHg vs.131 mmHg, *p*<0.001). BALF was collected on day 7 (*IQR* 2–13) of mechanical ventilation among patients with mild ARDS and day 5 (*IQR* 3–8) in those with moderate/severe ARDS. There was no significant difference between the proportion of microbiologically-confirmed VAP among patients with mild compared to moderate/severe ARDS (14% vs. 32%, *p* = 0.6). The most common organism isolated was *Haemophilus influenzae* followed by methicillin-sensitive *Staphylococcus aureus*. In-hospital mortality was 14% among patients with mild ARDS and 42% among those with moderate/severe ARDS (*p =* 0.4).

**Table 1:** Baseline characteristics of ARDS patients stratified by ARDS severity.

	All ARDS	Moderate/Severe ARDS (PaO_2_:FiO_2_ ≤200)	Mild ARDS (PaO_2_:FiO_2_ 201–300)
	*n = 26*	*n = 19*	*n = 7*
**Age, years (median (IQR))**	65 (49–75)	67 (51–79)	56 (47–62)
**Male, n (%)**	15 (58)	11 (58)	4 (57)
**Race/Ethnicity, n (%)**			
** White**	22 (85)	18 (95)	4 (57)
** Black**	3 (12)	0 (0)	3 (43)
** Hispanic**	1 (4)	1 (5)	0 (0)
**Ventilator-associated pneumonia, n (%)**	7 (27)	6 (32)	1 (14)
**PaO**_**2**_**:FiO**_**2**_ **ratio (median (IQR))**	155 (105–203)	131 (97–156)	220 (219–238)
**Total duration of mechanical ventilation, days (median (IQR))**	12 (9–20)	12 (9–17)	18 (10–27)
**In-hospital death, n (%)**	9 (35)	8 (42)	1 (14)

**Fig 1:**
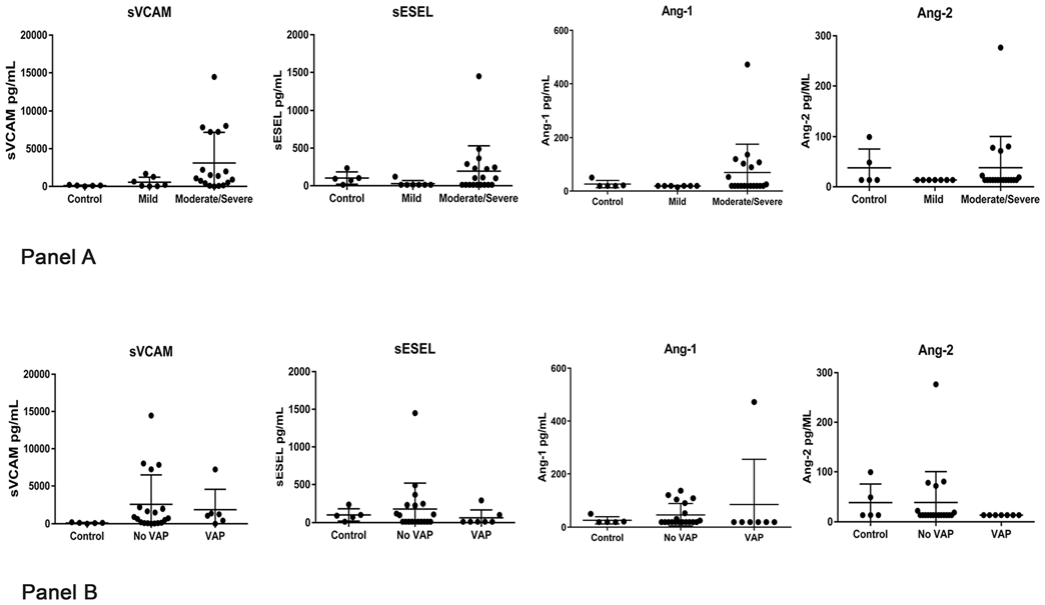
Levels of endothelial activation biomarkers in bronchoalveolar lavage fluid, by ARDS severity and microbiologically-confirmed ventilator-associated pneumonia. **Panel A**: Bronchalveolar lavage fluid (BALF) biomarker levels stratified by presence and severity of acute respiratory distress syndrome (ARDS) (**Control vs. *ARDS*, *p = 0*.*03*, *Mild vs*. *Moderate/Severe ARDS p = 0*.*06*). **Panel B**: BALF biomarker levels stratified by presence of microbiologically-confirmed ventilator-associated pneumonia (VAP).

**Fig 2:**
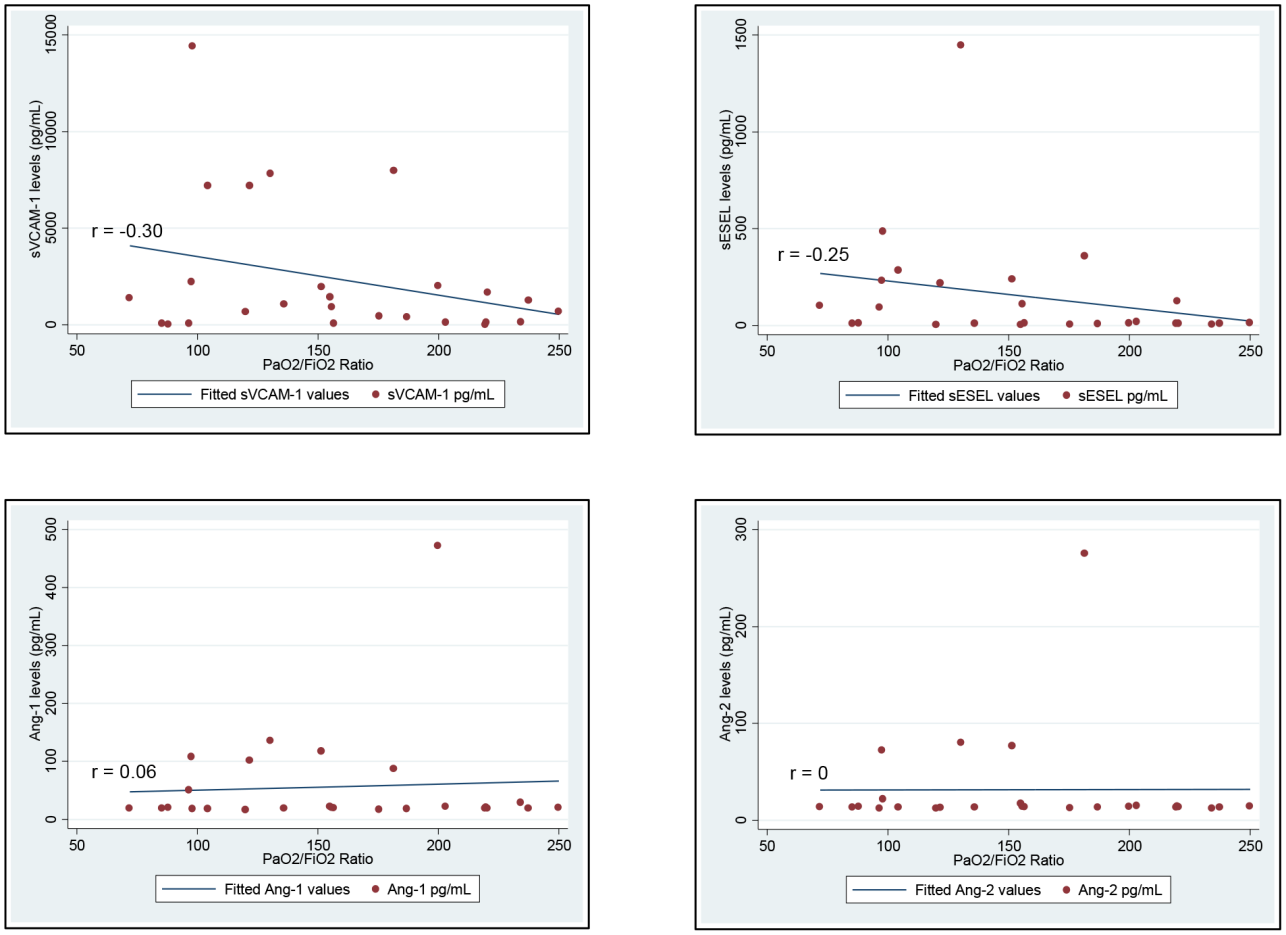
Levels of endothelial activation biomarkers in bronchoalveolar lavage fluid of ARDS patients by PaO_2_:FiO_2_ ratios.

Compared to five healthy controls, ARDS patients had significantly higher median BALF sVCAM-1 levels (985 pg/mL, *IQR* 134–2000, *vs* 119 pg/mL, *IQR* 86–124, *p =* 0.03; Fig 1, Panel A). Further, there was a trend toward greater BALF sVCAM-1 levels among patients with moderate/severe ARDS compared to those with mild ARDS (1395 pg/mL, *IQR* 418–7232 *vs* 209 pg/mL, *IQR* 42–1276, *p =* 0.06). Median BALF Ang-1, Ang-2 and sESEL concentrations did not differ significantly between ARDS patients and controls or by ARDS severity (Fig 1, Panel A and Table 2). When comparing continuous levels of BALF biomarkers with the PaO_2_:FiO_2_ ratio among ARDS patients, sVCAM-1 tended to be weakly inversely correlated with continuous PaO_2_/FiO_2_ (*r* = -0.30, *p =* 0.1); we detected no other associations between BALF biomarker levels and PaO_2_:FiO_2_ ratios (Fig 2). Additionally, there were no significant correlations between days elapsed prior to BALF collection and endothelial activation marker levels (sVCAM, *r =* 0.05, *p =* 0.8; sESEL, *r =* -0.06, *p =* 0.8; Ang-1, *r =* -0.13, *p =* 0.6; Ang-2, *r =* 0.20, *p =* 0.4).

**Table 2:** Levels of endothelial activation biomarkers (pg/mL) in bronchoalveolar lavage fluid of ARDS patients and controls.

	All ARDS	Moderate/Severe ARDS (PaO_2_:FiO_2_ ≤200)	Mild ARDS (PaO_2_:FiO_2_ 201–300)	Controls
	*n = 26*	*n = 19*	*n = 7*	*n = 5*
**sVCAM, median (IQR)**	985 (134–2000)*	1395 (418–7232)**	208 (42–1276)	119 (86–124)
**sESEL, median (IQR)**	12 (12–222)	100 (12–244)	12 (12–12)	93 (72–103)
**Ang-1, median (IQR)**	20 (20–53)	20 (20–103)	20 (20–20)	20 (20–21)
**Ang-2, median (IQR)**	14 (14–14)	14 (14–22)	14 (14–14)	14 (14–49)

*Control vs. ARDS, χ^2^ *p =* 0.03,

**Mild vs. Moderate/Severe ARDS χ^2^ *p =* 0.06

Median BALF biomarker levels also did not differ significantly between patients with and without VAP diagnoses (Fig 1, Panel B). The associations described above were similar when we restricted analyses to patients who did not have microbiologically-confirmed VAP (Table 3).

**Table 3:** Levels of endothelial activation biomarkers (pg/mL) in bronchoalveolar lavage fluid restricted to patients *without* microbiologically-confirmed VAP and controls.

	All ARDS (without VAP)	Moderate/Severe ARDS (PaO_2_:FiO_2_ ≤200)	Mild ARDS (PaO_2_:FiO_2_ 201–300)	Controls
	*n = 19*	*n = 13*	*n = 6*	*n = 5*
**sVCAM, median (IQR)**	748 (94–2203)	1515 (515–7238)	147 (42–649)	119 (86–124)
**sESEL, median (IQR)**	12 (12–229)	109 (12–244)	12 (12–12)	93 (72–103)
**Ang-1, median (IQR)**	20 (20–89)	25 (20–103)	20 (20–20)	20 (20–21)
**Ang-2, median (IQR)**	14 (14–22)	14 (14–72)	14 (14–14)	14 (14–49)

We did not detect significant associations between BALF biomarker levels and the clinical outcomes of either duration of mechanical ventilation (sVCAM, *r =* -0.21, *p =* 0.3; sESEL, *r =* -0.16, *p =* 0.5; Ang-1, *r =* -0.15, *p =* 0.5; Ang-2, *r =* 0.05, *p =* 0.8) or in-hospital deaths in our cohort (Table 4).

**Table 4:** Levels of endothelial activation biomarkers (pg/mL) in bronchoalveolar lavage fluid by in-hospital death.

	In-hospital death		
	Yes	No	*p-*value
	*n = 9*	*n = 17*	
**sVCAM, median (IQR)**	1395 (418–2000)	7748 (94–2000)	0.6
**sESEL, median (IQR)**	103 (12–244)	12 (12–121)	0.2
**Ang-1, median (IQR)**	20 (20–53)	20 (20–32)	0.9
**Ang-2, median (IQR)**	14 (14–14)	14 (14–14)	0.9

## Discussion

In this study, we found that BALF sVCAM-1 levels measured several days into the course of ARDS were significantly elevated compared to levels in healthy controls. We also analyzed endothelial activation biomarkers by ARDS severity and found that BALF sVCAM-1 concentrations tended to be higher among patients with moderate/severe ARDS compared to mild ARDS.

Our findings build upon the results of a prior study that found no difference in BALF sVCAM-1 levels between mechanically ventilated patients with and without ARDS [8]. Importantly, in that cohort, BALF samples were collected within two hours of intensive care unit admission, markedly earlier than in our study and likely relatively earlier in the pathogenesis of ARDS. Early sampling of the alveolar space may not fully reflect the complex contribution of endothelial activation to the pulmonary endothelial-epithelial (i.e., vascular-alveolar) barrier dysfunction in ARDS. Upon activation, endothelial cells upregulate cell surface adhesion molecule expression, and soluble isoforms, such as sVCAM-1, are subsequently shed. These isoforms accumulate in the plasma and alveolar fluid, modulating the inflammatory response [20] which is likely to impact pulmonary endothelial-epithelial barrier dysfunction and ARDS severity. Further studies exploring early versus late sampling strategies are necessary to elucidate the kinetics, ideal sampling frame and test characteristics of BALF sVCAM-1 as a biomarker of ARDS.

VCAM-1 is an endothelial adhesion molecule that regulates leukocyte recruitment during inflammation. VCAM-1 is cleaved from the endothelial cell surface to form soluble sVCAM-1. sVCAM-1 has biologic activity and can block adhesion of inflammatory cells to the endothelium and function to dampen the inflammatory response [20]. Regulation of VCAM-1 expression and sVCAM-1 shedding in ARDS, especially in the setting of VAP, is not extensively studied. Data available from patients with sepsis, the most common cause of ARDS, suggest that higher sVCAM-1 levels correlate with severity of sepsis [21]. Other studies, however, report an association between mid-range levels of soluble adhesion molecules and worse outcomes [20]. The blunted elevations in soluble isoforms were attributed to inadequate or aberrant shedding, and may lead to unresolved inflammation and persistent endothelial activation [20]. Interestingly, a post-mortem study demonstrated over-expression of endothelial cell surface VCAM-1 in the pulmonary vessels of patients who died from ARDS [22], but BALF sVCAM-1 levels were not measured in that study. It is unclear whether our findings of elevated BALF sVCAM-1 amongst ARDS patients reflect expected levels of shedding or, conversely, a dysregulated pathophysiologic mechanism, especially in the presence of concomitant VAP.

We detected no significant differences in sESEL, Ang-1 or Ang-2 BALF concentrations between patients with ARDS who were undergoing evaluation for clinically suspected VAP and healthy volunteers. In a study of patients with chronic alcohol abuse, although BALF sESEL levels were elevated, levels did not differ significantly between patients with and without ARDS [9]. Also, previous investigators demonstrated elevated Ang-2 and decreased Ang-1 levels in the BALF of patients with hyperoxia-induced acute lung injury [5]. However, in our study, we did not identify appreciable differences in the levels of Ang-2 and Ang-1 in BALF, suggesting that alveolar accumulation of angiopoietin may not be uniform across types of lung injury. We also did not identify differences in median BALF biomarker levels between patients with and without microbiologically-confirmed VAP. The small sample size of our study may have impacted our ability to detect associations between the studied BALF biomarker levels and ARDS. Further, our study was not sufficiently powered to detect differences in clinical outcomes, such as ventilator days or in-hospital mortality associated with BALF biomarker concentrations.

Although we did not measure levels of cytokines in BALF, inflammatory cytokines have been implicated in ARDS pathogenesis, severity and outcomes, and mediate the expression of endothelial activation biomarkers [2, 23]. For instance, VCAM-1 expression on endothelial cell surfaces has been shown to be induced by IL-4 and IL-13 [24, 25] as well as other inflammatory exposures [26], and our finding of elevated sVCAM-1 in BALF is consistent with these published data. Conversely, the expression of E-selectin is downregulated over time by IL-4 and IL-13 [27], supporting our finding that E-selectin was not markedly elevated in the BALF of patients with ARDS collected a median of 5 or 7 days after intubation. Further, among other inflammatory cytokines, IL-4 has been described to rise in BALF over the course of ARDS progression, particularly in the setting of sepsis [28].

Our study has several other limitations, and, accordingly, detected associations or the lack thereof should be interpreted with caution. Firstly, only patients with ARDS and clinical suspicion for VAP underwent bronchoalveolar lavage. This may have introduced bias if a process mimicking VAP affects BALF endothelial activation biomarkers. However, we found no significant differences in BALF biomarker concentrations between patients who did and did not have microbiologically-confirmed VAP. Secondly, BALF samples were collected at a single time point, owing to the pragmatic nature of this study, which analyzed biomarkers collected from clinically indicated bronchoscopies. Thirdly, the use of BALF rather than undiluted pulmonary edema fluid for our analyses may have accounted for some of the differences observed in our study compared with prior studies; we were unable to calculate the dilution factor of BALF. Finally, although we could not exclude masking of proteins by binding to matrix components, we have not encountered difficulties in prior measurements of select proteins in the BALF of patients with ARDS [29–31].

Nonetheless, our study’s noteworthy strength is that we analyzed biomarkers of endothelial activation from the BALF of patients with ARDS. Findings from samples obtained directly from the alveolar space may provide new insights specific to the effects of endothelial activation in the lungs in the face of the often systemic involvement of ARDS. Recent data suggest that bronchoalveolar sampling for inflammatory biomarkers may better predict VAP compared to serum cytokine analysis [32], and this may extend to endothelial activation markers in ARDS.

## Conclusions

We found that BALF sVCAM-1 concentrations were significantly elevated in ARDS and tended to be greater in moderate/severe compared to mild ARDS. Our findings add to the growing evidence supporting the concept that endothelial activation plays an important mechanistic role in the pathogenesis of ARDS. Further studies are necessary to understand the role of endothelial activation in the pathogenesis of ARDS in VAP and other conditions.

## Supporting Information

S1 Data Raw data collected and interpreted for this study. (XLSX)
